# Supplementary material for: Causal Relationship between Aging and Anorexia Nervosa: A White-Matter-Microstructure-Mediated Mendelian Randomization Analysis
Source: Biomedicines. 2024 Aug 16;12(8):1874. doi: 10.3390/biomedicines12081874 (PMC11351342; doi:10.3390/biomedicines12081874)
Supplement: Supplementary file 1 [file biomedicines-12-01874-s001.zip › Supplementary Figure/Figure S4/Singe SNP plot of MR analysis for TL to ubm-b-1957.pdf]

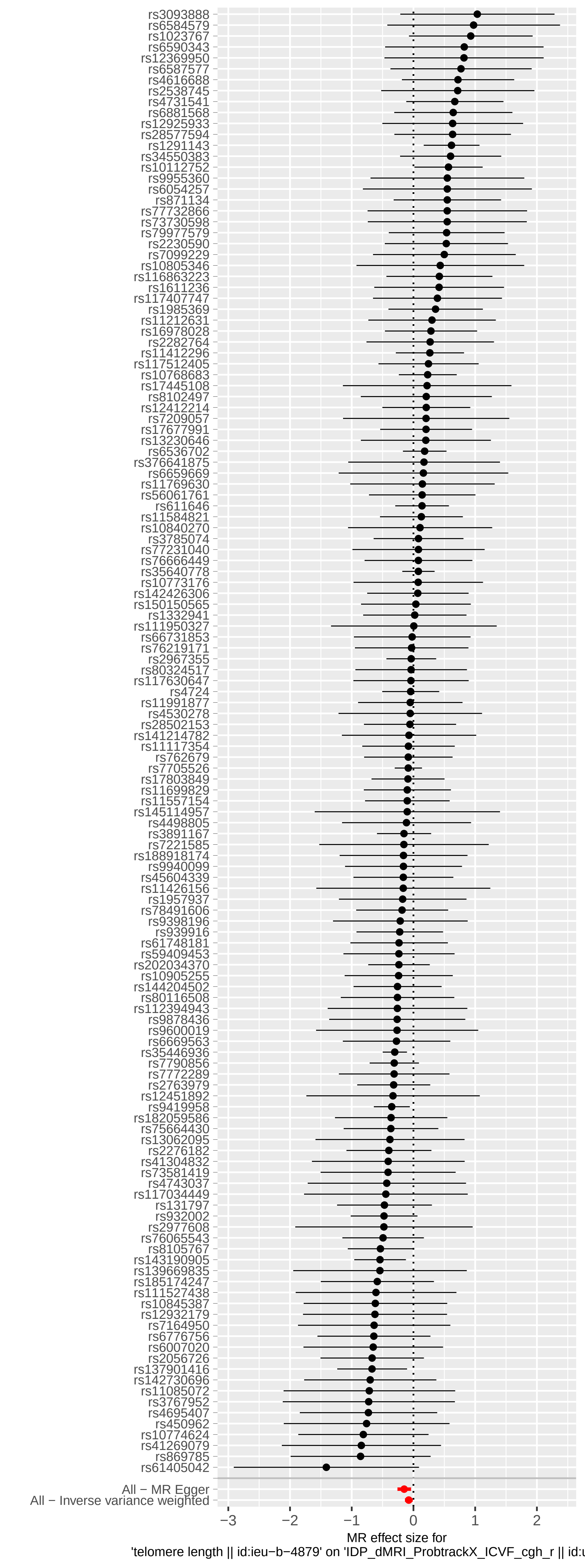
rs3093888
rs6584579
rs1023767
rs6590343
rs12369950
rs6587577
rs4616688
rs2538745
rs4731541
rs6881568
rs12925933
rs28577594
rs1291143
rs34550383
rs10112752
rs9955360
rs6054257
rs871134
rs77732866
rs73730598
rs79977579
rs2230590
rs7099229
rs10805346
rs116863223
rs1611236
rs117407747
rs1985369
rs11212631
rs16978028
rs2282764
rs11412296
rs117512405
rs10768683
rs17445108
rs8102497
rs12412214
rs7209057
rs17677991
rs13230646
rs6536702
rs376641875
rs6659669
rs11769630
rs56061761
rs611646
rs11584821
rs10840270
rs3785074
rs77231040
rs76666449
rs35640778
rs10773176
rs142426306
rs150150565
rs1332941
rs111950327
rs66731853
rs76219171
rs2967355
rs80324517
rs117630647
rs4724
rs11991877
rs4530278
rs28502153
rs141214782
rs11117354
rs762679
rs7705526
rs17803849
rs11699829
rs11557154
rs145114957
rs4498805
rs3891167
rs7221585
rs188918174
rs9940099
rs45604339
rs11426156
rs1957937
rs78491606
rs9398196
rs939916
rs61748181
rs59409453
rs202034370
rs10905255
rs144204502
rs80116508
rs112394943
rs9878436
rs9600019
rs6669563
rs35446936
rs7790856
rs7772289
rs2763979
rs12451892
rs9419958
rs182059586
rs75664430
rs13062095
rs2276182
rs41304832
rs73581419
rs4743037
rs117034449
rs131797
rs932002
rs2977608
rs76065543
rs8105767
rs143190905
rs139669835
rs185174247
rs111527438
rs10845387
rs12932179
rs7164950
rs6776756
rs6007020
rs2056726
rs137901416
rs142730696
rs11085072
rs3767952
rs4695407
rs450962
rs10774624
rs41269079
rs869785
rs61405042
All − MR Egger
All − Inverse variance weighted
−3
−2
−1
0
1
2
MR effect size for
'telomere length || id:ieu−b−4879' on 'IDP_dMRI_ProbtrackX_ICVF_cgh_r || id:u
